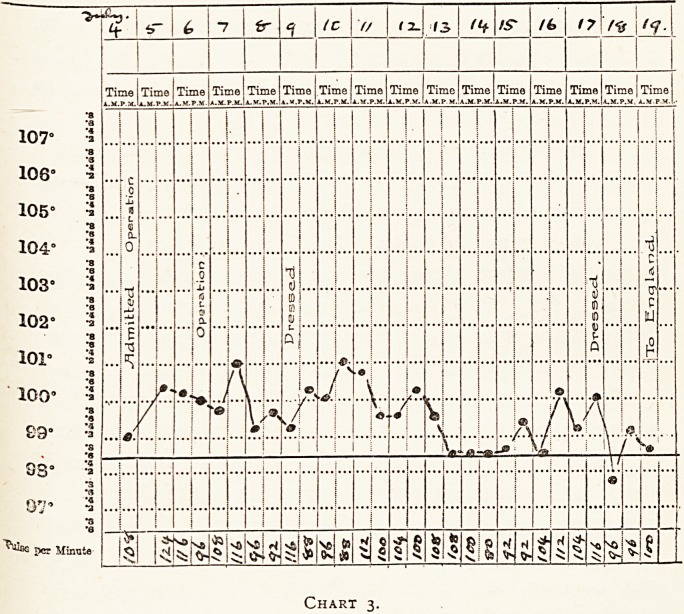# Some Aspects of British Surgery in France

**Published:** 1916-12

**Authors:** James Swain

**Affiliations:** Lt.-Col. R.A.M.C.; Consulting Surgeon to the Southern Command


### SOME ASPECTS OF BRITISH SURGERY IN FRANCE.

BY

JAMES SWAIN, M.S., M.D. London., F.R.C.S.,

*Lt.-Col. R.A.M.C.; Consulting Surgeon to the Southern Command.*

---

It may be of interest to those at home to know some of the principles of treatment applied to cases of gunshot wounds within the first few days of their occurrence, for the problems which confront the surgeon abroad are very different from those which occur in England. This is largely owing to the fact that practically all wounds are in an acutely septic condition, a condition of urgent and paramount importance, which necessitates the use of free incisions for the relief of tension and adequate drainage of the wounds. Much of the work at the casualty clearing stations and base hospitals consists in energetically combating this sepsis, and without the results thus obtained many of the cases would die of acute toxic poisoning, or would suffer more severely from the extension of the septic mischief during their transference to England.

The treatment of well-marked "gas gangrene" is the same in all the hospitals I visited while Acting-Consulting Surgeon in France, viz. circular amputation without flaps. It is impossible to enter here upon the controversy which has arisen with regard to this form of treatment. It must suffice to say that any form of flap is more than likely to lead to a further development of the bacillus perfringens with extension of gangrene, and I am in agreement with those who consider the flapless amputation for gas gangrene to be a life-saving operation.

The "straight" amputation should be done above the gas-infected tissues; but this is sometimes impracticable, and I have seen cases recover where the line of amputation necessarily passed through the infected area.

It is of the greatest importance not to confuse gas gangrene with the local varieties (gaseous cellulitis, etc.) of infection with gas-forming bacilli.

The treatment of these local infections differs according to the views of the surgeon. Some operators practically excise the whole wound, so as to expose healthy muscle and skin. Such an operation in a deep wound with widely-lacerated muscles must cause considerable shock. One would also suppose that the exposure of such a large fresh surface to infection might lead to serious toxic absorption, but it is said that this has not been found to result. By others these cases are successfully treated by drainage and free incision only, care being taken that no tension or "pocketing" is allowed to remain. Sometimes a deeply-invaded muscle is removed, but there is no such excision of the whole wound as previously referred to. It seems clear, therefore, that it is unnecessary to submit the patient to the shock of a free excision of wounds accompanied by local infection with gas-forming bacilli. A case in point is the following:—

Private W. was wounded on July 1st, and admitted to hospital on July 4th with a shell wound and extensive spreading sepsis of the upper part of the right thigh reaching on to the abdomen. The general condition was most unsatisfactory, and gave rise to anxiety. A large shell fragment was removed from a stinking abscess cavity full of gas. There was sloughing of the subcutaneous and intermuscular tissues. Very free multiple incisions were made, and the wounds were dressed with salt tablets. Two days later further incisions were made. The condition rapidly improved. On July 17th the wounds were healthily granulating, and on July 19th he was sent to England.

As regards the treatment of septic wounds generally there is a considerable divergence of opinion as to what is best after the inevitable incisions for the relief of tension. At some hospitals the wounds are lightly packed with gauze wrung out of five per cent. saline solution and covered with wool and bandage, at others eusol is used, sometimes mixed with equal parts of ten per cent. saline solution, in a similar manner. In both cases the wounds are dressed once or twice daily, and irrigated at the time of dressing with the same solution as that used for wetting the gauze.

At other hospitals, again, the treatment of septic wounds consists in packing them with gauze, enclosing tablets of salt, each tablet containing sixteen grains of salt and three grains of citrate of potash. Large numbers of these tablets are used by placing them on two to four layers of gauze about three or four inches wide, and twisting the gauze so as to enclose the tablets. The wound is then covered with dry gauze, wool and bandage, or dressed with eusol or saline solution as referred to above. Pain is caused for about an hour, and the tablets are not wholly dissolved for about forty-eight hours. After being dressed in this way the wound is left by some surgeons for a week or longer according to circumstances.

The advocates of this last method maintain that it is quite unnecessary to dress these cases frequently, and that by leaving them alone you save the patient from much pain and shock. Cases are commonly dressed after an interval of about a week from the primary dressing, as by this time the gauze easily floats off with the pus which has accumulated beneath. If, however, during this period the temperature rises or the pulse is accelerated, and particularly if the wound becomes painful, a further exploration would be made and any " pocketing " or tension relieved.

It is needless to say that decomposition occurs, but the patients appear to prefer to put up with the foul odour of putrefaction rather than to bear the pain of frequent dressings. The smell can be partly kept under by more frequently changing the outer dressings, without interfering with the part of the dressing next to the wound; for those who prefer this form of treatment regard the frequent removal of the whole dressing as deleterious to both wound and patient. As examples of infrequent dressing of wounds surrounded by stinking pus without appreciable harm resulting the following cases may be cited :—

CHART I.

Private M. was wounded on July 8th and admitted to a base hospital on July 13th. The note sent with him from the casualty clearing station stated that he had a compound fracture of both bones of the forearm; that the arm was very tense on admission, and threatened gangrene was relieved by incisions; that the wounds of the shoulder were very dirty, and that the case required "frequent dressing." On admission to the base hospital the patient looked very ill, there were severe multiple wounds of the left arm and forearm with extensive sloughing, and fracture of both bones of the forearm. The wounds were dressed with gauze and ten per cent. saline, and they were not dressed again until July 22nd (nine days after admission), when the patient was transferred to England. The frequent dressings previously employed had been very painful, and the patient expressed his preference for the form of treatment adopted at the base hospital.

CHART 2.

Private M. was wounded on July 23rd, and admitted to hospital on July 25th with a bullet wound which traversed the shoulder-joint from front to back, and associated with comminuted fracture of the head of the humerus. The wound was packed with salt tablets and gauze, posteriorly, down to the joint, and not dressed again till July 30th. On August 1st he was sent to England.

CHART 3.

Private W., wounded on July 1st. This is the case of localised gas infection of the thigh already referred to. The wounds were not dressed between July 9th and July 17th, when they were found to be healthily granulating. Some dermatitis of the back of the thigh had been caused by the irritation of the pus, but this had practically disappeared when he went to England two days later.

To leave wounds bathed in decomposing pus seems abhorrent to those who have been educated in modern aseptic principles during times in which septic wounds rarely occurred, but in military surgery in the present war almost all wounds are septic, and the conditions more nearly resemble those which obtained before the days of Lister.

The salt pack used in these cases where the dressing is left unchanged for long periods is regarded by its advocates as an essential part of the treatment, and probably this is so, for the salt produces a watery flow through the wound which keeps the surface moist, and as a mild antiseptic tends to inhibit the growth of organisms.

I am of opinion that wounds granulate more quickly when dressed once or twice daily with eusol or other antiseptic than when left for five or six days or more, without dressing, after putting in salt packs. This is probably owing to the deleterious effect of pus on the newly-formed granulation tissue; for, owing to the proteolytic ferments of its causative organisms, pus may irritate the granulation tissue and thus delay the initial process of repair.

It must, however, be conceded that this method of infrequent change of dressings has advantages in the early stages of treatment of some cases. Wounds are often large, deep, and extremely painful owing to the inflammation accompanying acute sepsis. The dressing of these wounds produces considerable shock, and to dress them frequently might easily turn the scale against a bad case. To leave the dressing unchanged for several days may save the lives of such patients, and certainly saves much pain under conditions where time and a plentiful supply of nurses, both of which are essential if frequent dressings are undertaken, may be wanting. For purposes of transport it is particularly useful to know that wounds can be packed with salt, and then left undisturbed for several days without serious consequences. After the first week, when the wound is freely suppurating and the dressings float off easily, there is not the same advantage in leaving the dressing unchanged for a long period.

One might suppose that the advanced decomposition in these wounds would give rise to pyæmia and secondary hemorrhage. That some cases should die of toxæmia is inevitable in the acute septic conditions which occur in the present war; but pyæmia does not seem to occur, and secondary hemorrhage is apparently no more common than amongst septic wounds treated by more frequent dressing

Though possessing some advantages in the directions I have already indicated, the method is not ideal, and it may be worth bearing in mind that Dr. Carrel has devised a means by which wounds may be more or less continuously irrigated through perforated rubber tubes without disturbing the dressing.

Many operations are performed under spinal anæsthesia or local anæsthesia. For the former stovain is used, and this is occasionally supplemented by a very slight general anæsthesia in nervous patients. Operation under stovain is frequently unattended by any marked degree of shock, and I have amputated in the upper third of the thigh by this method of producing anæsthesia without any appreciable effect on the pulse of the patient. Spinal anæsthesia with stovain is useful in war surgery, for many cases are unfit for deep general anæsthesia. On the other hand, the patient may exhibit grave symptoms, and die of respiratory failure with cyanosis and widely-dilated pupils. I have seen a patient with these urgent symptoms recover immediately on the withdrawal of some cerebro-spinal fluid by lumbar puncture. A preliminary subcutaneous injection of strychnia is frequently used to prevent this tendency to respiratory embarrassment. For local anæsthesia one per cent. of novocain with adrenalin is used, anæsthesia being produced in a few minutes. Most operations upon the head are performed by this method, which is also useful for the common practice of completely excising septic tracks for the purpose of obtaining union by first intention.

Reference must be made to the "electro-vibreur" of

Professor Bergonie. This useful instrument practically consists of an electro-magnet suspended from a frame and charged from the main electric supply. When it is brought near iron (shrapnel casing, ferro-nickel of German rifle bullet, etc.) a rapid vibration of the metal is produced, which can be felt by the hand placed over the part in which the substance is buried. The point of greatest intensity of the vibration marks the locality of the foreign body. If the metal is too deep for the vibrations to be felt by the hand they may be heard with the stethoscope. When work is heavy and there is little time for exact localisation by X-rays this machine is invaluable, and even at operations in ordinary times it supplements the skiagram or allows one to dispense with the screen. The method is valueless if the metal is fixed, and of course only iron is attracted.

### MEDICINE AND SURGERY IN MESOPOTAMIA.

BY

CAPT. CAREY F. COOMBS, R.A.M.C. (T.).

## Figures and Tables

**Chart 1. f1:**
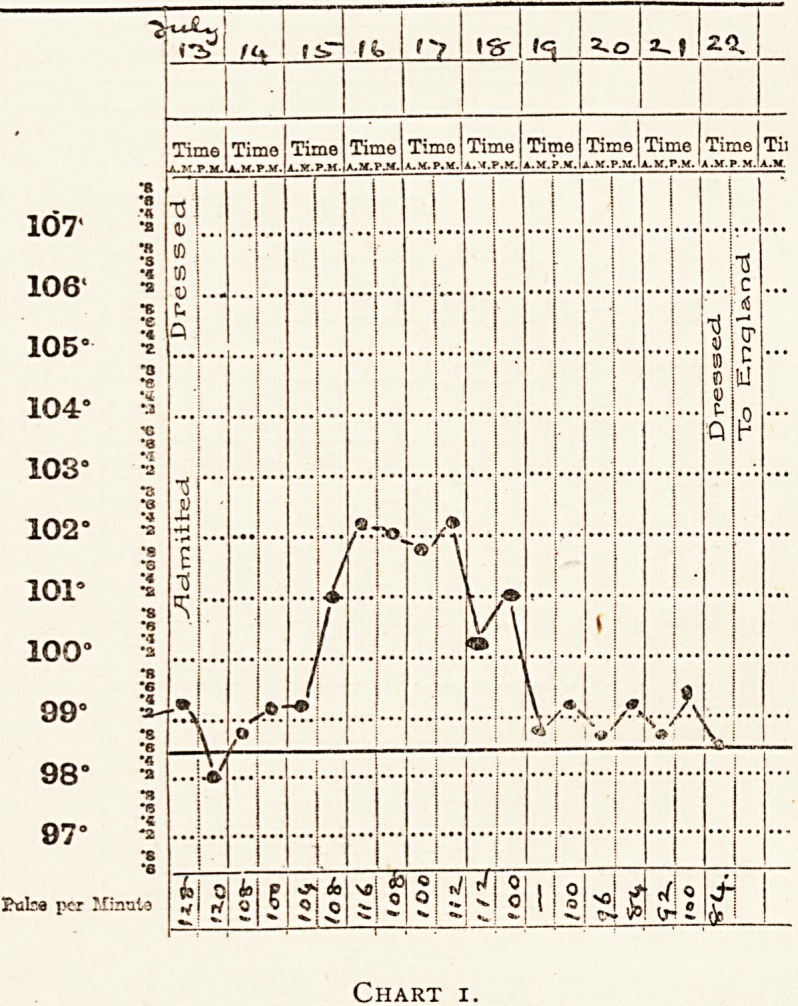


**Chart 2. f2:**
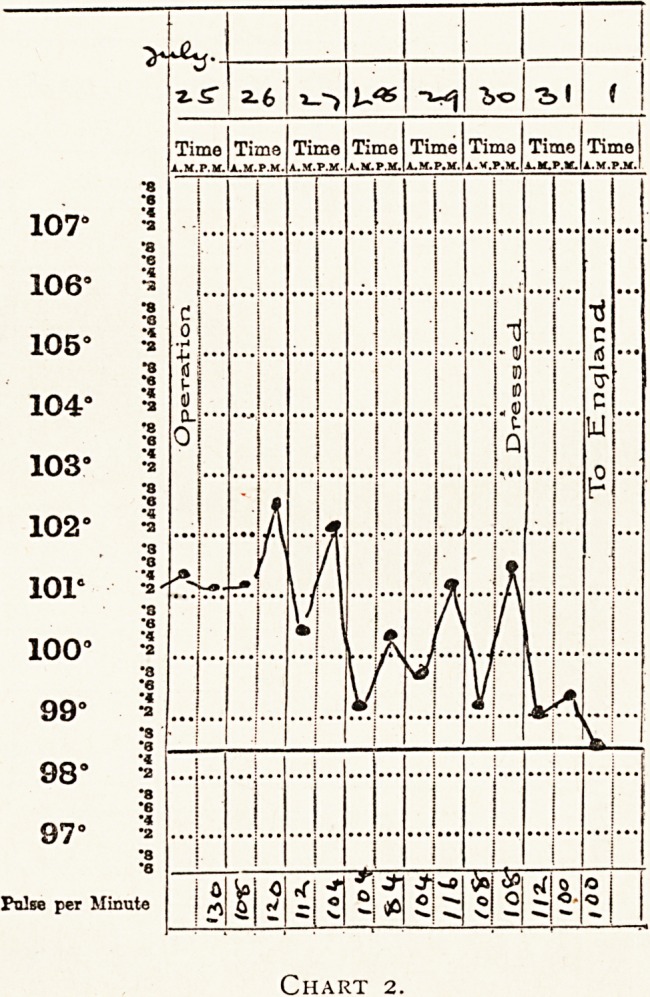


**Chart 3. f3:**